# Development of a hybrid framework for inventory leanness in Technical Services Organizations

**DOI:** 10.1371/journal.pone.0247144

**Published:** 2021-02-19

**Authors:** Khurram Rehmani, Afshan Naseem, Yasir Ahmad, Muhammad Zeeshan Mirza, Tasweer Hussain Syed

**Affiliations:** Department of Engineering Management, College of Electrical and Mechanical Engineering, National University of Sciences & Technology (NUST), Islamabad, Pakistan; International Centre for Integrated Mountain Development (ICIMOD), Kathmandu, NEPAL

## Abstract

Inherent uncertainties in demand and supply make it problematic for supply chains to accomplish optimum inventory replenishment, resulting in loss of sales or keeping excessive inventories. To cope with erratic demands, organizations have to maintain excessive inventory levels, sometimes taking up to one-third of an organization’s annual budget. The two most pressing concerns to handle in inventory management are: how much to order and when to order. Therefore, an organization ought to make the correct and timely decisions based on precise demand information to avoid excessive inventory accumulation resulting in enhanced competitive advantage. Owing to the significance of inventory control and analysis, this paper reports on developing and successfully implementing a hybrid framework for optimum level inventory forecasting in Technical Services Organizations. The proposed framework is based on a case study of one of Pakistan’s leading Technical Services Organization. The paper presents a statistical analysis of historical data and a comprehensive fault trend analysis. Both these analyses set a solid foundation for the formulation of a comparative analysis matrix based upon price and quantity based analysis of inventory. Finally, a decision criterion (Forecasting Model) is proposed using three primary forecasting techniques with minimum error calculations. The study’s finding shows a forecast error of 142.5 million rupees in the last five years, resulting in the accumulation of more than 25 thousand excessive inventory stock. Application of price and quantity based analysis identifies that 65% of the annual budget is significantly dependent upon only 9% (in terms of quantity) of "High Price and Small Quantity" Items (HS). These HS items are forecasted through three different forecasting methods, i.e., Weighted Moving Average, Exponential Smoothing, and Trend Projection, with Minimum Absolute Deviation to significantly reduce the forecasting error while predicting the future required quantity. The research work aims to contribute to the inventory management literature in three ways. First, a new comparative analysis matrix concept for identifying the most critical items is introduced. Second, a Multi-Criteria Forecasting Model is developed to capture a wide range of operations. Third, the paper suggests how these forecasting criteria can be integrated into a single interactive DSS to maintain optimum inventory level stock. Even though the DSS framework is based on data from a single organization, the application is expected to manage inventory stock in a wide range of manufacturing and services industries. This study’s proposed hybrid framework is the first of its kind that encapsulates all four dimensions of inventory classification criteria, forming a multi-criteria hybrid model within a DSS framework.

## 1. Introduction

Given the significance of inventories as valuable strategic resources for organizations, inventory management has always been one of the dominant areas of investigation in the operations management (OM) literature [1, 2]. The methodology to inventory management that has taken the most extensive account in recent decades is the lean inventory philosophy that sights excess inventories as waste and emphasizes fostering inventory efficiency in firms [3]. Likewise, inventory control relies intensely on future demand forecasting, whereas the literature assumes that demand distributions are known [4]. This implies that estimates are substituted directly for the unknown parameters, resulting in inadequate safety stocks, stock-outs, low services, and high costs. In contrast, the inventory control literature reveals a clear demarcation between demand forecasting and inventory decision making. Since researchers like Harris [5] and Lowe & Schwarz [6] established the economic order quantity models, various other frameworks have also been developed, using erratic review structures [7], cost frameworks [8], and demand characteristics [9]. While evaluation is crucial in forecasting analysis, often this is constrained to looking at the forecast accuracy, which is anticipated to be a reasonable proxy for the decisions supported by the forecasts [10].

Most (small/medium-sized) organizations currently utilize inventory control models either through particular inventory management software or universal ERP software. Nevertheless, most inventory models/frameworks generally depend upon a high degree of future demand distribution that, unfortunately, never occurs in practice. Although managing optimal forecasting of intermittent demands has received considerable research, the interface between demand forecasting and decision making remains ambiguous.

On the other side, there is a growing consensus that organizations can achieve higher inventory efficiency from effective decision support systems (DSS), ensuring a sizeable reduction in dead and inactive inventories [11]. An effective Decision Support system shadows a magnanimous effect on any organization’s efficiency. It encapsulates four significant factors related to an effective inventory control system, i.e., material flow, information flow, procurement, and delivery [12]. The "most expensive" material is the one that is "not available" when it is needed, and the "second most expensive" material is the available one but "not needed" [13]. Therefore, inventory management’s prime objective is to devise an effective system that can optimize the stocks so that material is kept at a minimum possible capital input without disturbing the output and ensuring increased profitability and productivity. The best supply organizations equipped themselves with the most sophisticated and practical analytical tools to bring down the inventory levels by 20 percent to 50 percent, which yields in savings for years" [13].

Unfortunately, the historical data of inventory stocks held by the leading Public Sector’s Technical Services Organization (TSOs) of Pakistan reveals a considerable amount of dead/inactive inventory piling up every year due to inefficient procurement and ineffective forecasting techniques. The current paper develops a multistage hybrid framework for optimum inventory forecasting in the Public Sector’s engineering organization of Pakistan to address this anomaly. A case study of one of the leading engineering organizations is carried out using primary data between 2015 to 2019. The studied organization deals with the repair, maintenance, and modifications of Surveillance /Telecom and IT equipment.

## 2. Theoretical background

The literature indicates numerous studies focusing on different facets of spare parts demand forecasting and inventory control, including items classification [14], time bucket selection [15], demand forecasting models [16], lead-time demand distribution [17], and parameter revision frequencies [4]. Most of the less recent research on inventory management focuses on traditional inventory control models. Researchers of that era have either assessed conventional inventory control models under specific conditions or combined additional deliberations into established models [18]. The basic (Q, r) inventory control model was introduced by Harris [5], which allowed an organization to place orders of size (Q) whenever its inventory position reaches a re-order point (r). Carrying that basic concept further ahead, the logistics literature has extended the (Q, r) approach in several other aspects. For example, researchers have considered certain additional factors, like transportation [19], buyer/seller relationships [20], quality considerations, short lead times and emergency conditions, etc. [18]. and have further evaluated the approach under particular demand and lead-time distributions [21]. Recent research on demand forecasting and inventory control focuses on specific methods such as implementing Croston’s method and its subsequent modifications to inventory demand forecasting [22]. Numerous collaborative inventory management models tend to focus on the inventory decisions made by organizations participating in different cooperative programs, such as CRP, ECR, QR, and VMI. However, collaboration is a decision-making process that includes inter-dependent firms [23, 24].

In recent research, parallel to the forecasting models and DSS, various advanced and state of the art software and programs have been developed to optimize "Critical Spare Parts" provisioning like *SAP - SCM*, *Fishbowl Inventory*, *S2K Warehouse Management Software*, *Conga Novatus Contract Management*, *Slingshot Enterprise Business Suite*, *Infor Supply Chain Management*, *Oracle SCM Cloud*, *Pronto Xi*, *Oasis*, *JDA software*, etc. [3, 21]. However, these softwares are generally specific to the industry requirement. Likewise, various models have also been developed over the years using mono or multiple criteria. However, these models are either based on time series with demand variability or generally focused on intermittent demand forecasting with less emphasis on multi-criteria inclusion. Table 1 compares different forecasting models used in the literature regarding classification criteria, forecasting techniques, and model implementation.

**Table 1:** A comparative analysis of demand forecasting models.

Author	Classification Criteria				Forecasting Method	Mono Criteria	Multi-Criteria	Case Study with Implementation
	Parts Cost	Parts Criticality	Demand Volume	Demand Variability				
Makridakis et al. [25]	✔	✔		✔	1. Moving average, 2. Exponential smoothing 3. EWMA		✔	✔
					2. Exponential smoothing			
					3. EWMA			
Dolgui & Pashkevich [26]		✔		✔	Hybrid Model (Beta-binomial)		✔	
Hua et al. [27]			✔		Integrated Forecasting Method		✔	✔
Tibben‐Lembke & Amato [28]	✔				Fault Rate Analysis	✔		
Johnston & Boylan [29]				✔	Adjusted EWMA	✔		✔
Schultz [30]			✔		Croston’s method and modification	✔		✔
Chen et al. [31]		✔			Failure Rate forecasting	✔		
Jiang et al. [32]		✔		✔	Support Vector Machine Model		✔	
Kumar Sogi & Kumar Mittal [33]				✔	Time Series Forecasting		✔	
Kourentzes et al. [18]		✔	✔		Simulation Optimization	✔		

This study’s proposed hybrid framework is the first of its kind that encapsulates all four dimensions of classification criteria, forming a multi-criteria hybrid model within a DSS framework.

## 3. Case study

This section elaborates the case study results to assess the practical implementation of the proposed hybrid framework. Three Public Sector’s TSOs were approached to seek their approval for the current case study. The management of the targeted organizations was completely assured that the case study was solely envisioned for academic purposes, and the anonymity of the studied organizations would be ensured. Unfortunately, only one organization rendered its approval for the study, while two other firms regretted the case study’s request. The case study has been carried out in four sequential phases. At stage-1, the complete holding stock (inventory) statistical analysis was carried out to segregate the inventory as active, inactive, and dead inventory as per the set criteria. At stage-2, the fault-trend analysis was done to identify the active spare parts (inventory). At stage-3, price and quantity based analysis was performed using the 3x3 comparative analysis matrix technique. At stage-4, the identified spares were forecasted through the proposed DSS using three forecasting techniques with minimum error calculations.

## 4. Development of multi-criteria decision support system

**Fig 1:**
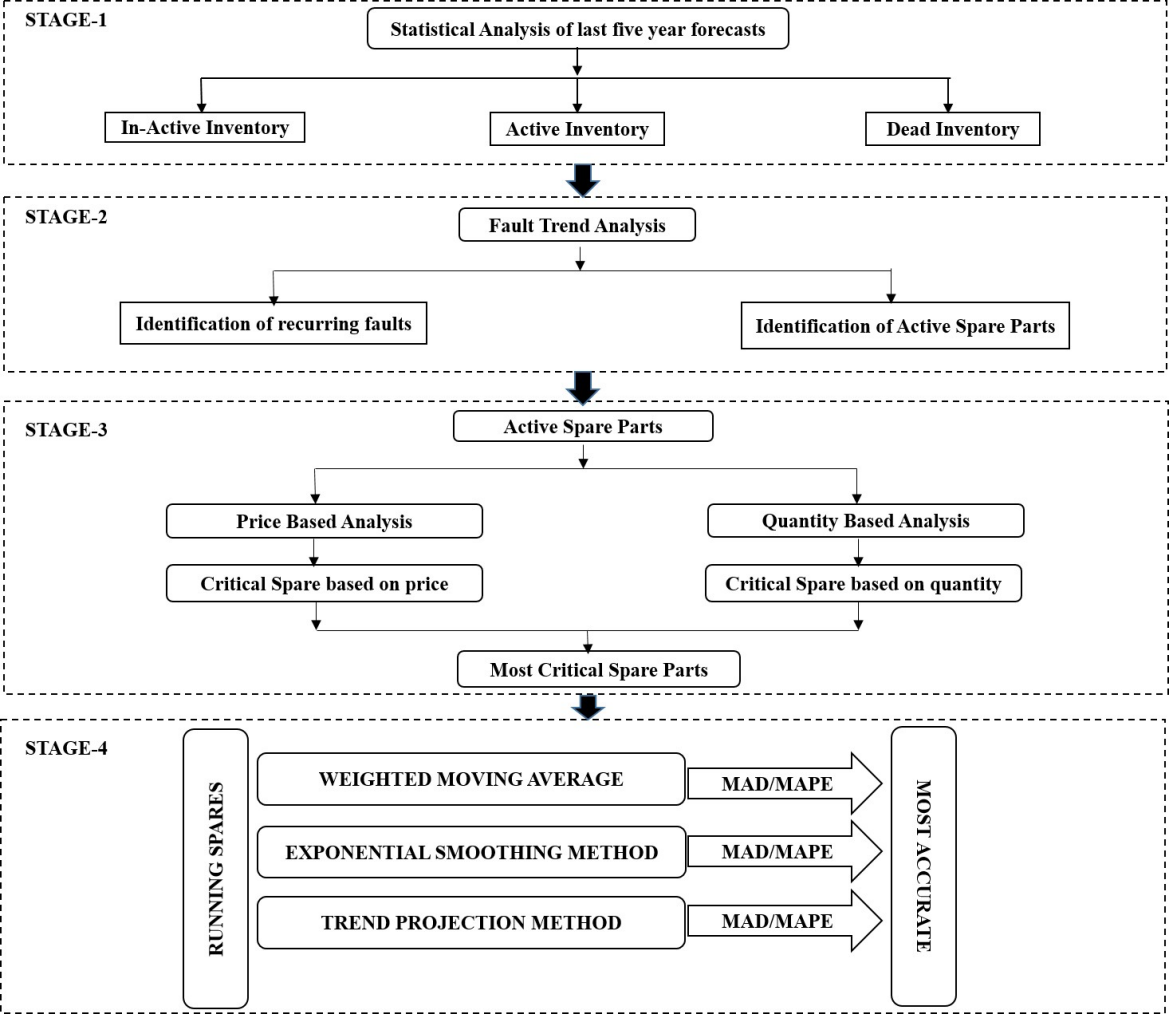
Theoretical framework of proposed DSS.

The sequence of action adopted in DSS development is depicted in Fig 1, where Stage-1 begins with collecting historical data and ends at stage-4 with a consolidated list of forecasted spares. The sequence is as follow:

### 4.1. Analysis of forecasted versus actual demands (stage-1)

Initially, primary data of forecasted (purchased) and utilized spares parts between 2015 to 2019 of studied Engineering Organization has been collected for quantitative analysis to have a fair idea of the demand and supply curve of various items and forecast errors of each year as shown in Table 2 below.

**Table 2:** Forecast errors in forecasted/utilized spares parts 2011–2015.

Year	Purchased Items	Purchased cost(PKR)	Utilized Items	Utilized Items cost	Forecast Error	% Utilization
					(C-E)	(D/B*100)
(A)	(B)	(C)	(D)	(E)	(F)	(G)
2015	6889	106 M	4594	64.9 M	**41.1 Mn**	67%
2016	4016	83.55 M	6102	52.3 M	**31.25 Mn**	152%
2017	5761	69.8 M	4983	57.8 M	**12 Mn**	86%
2018	7122	82.5 M	4086	42.6 M	**39.9 Mn**	57%
2019	3588	82.1 M	5008	63.4 M	**18.7 Mn**	140%
**Total**	**27376**	**423.95 M**	**24773**	**281 M**	**142.95**	

**Fig 2:**
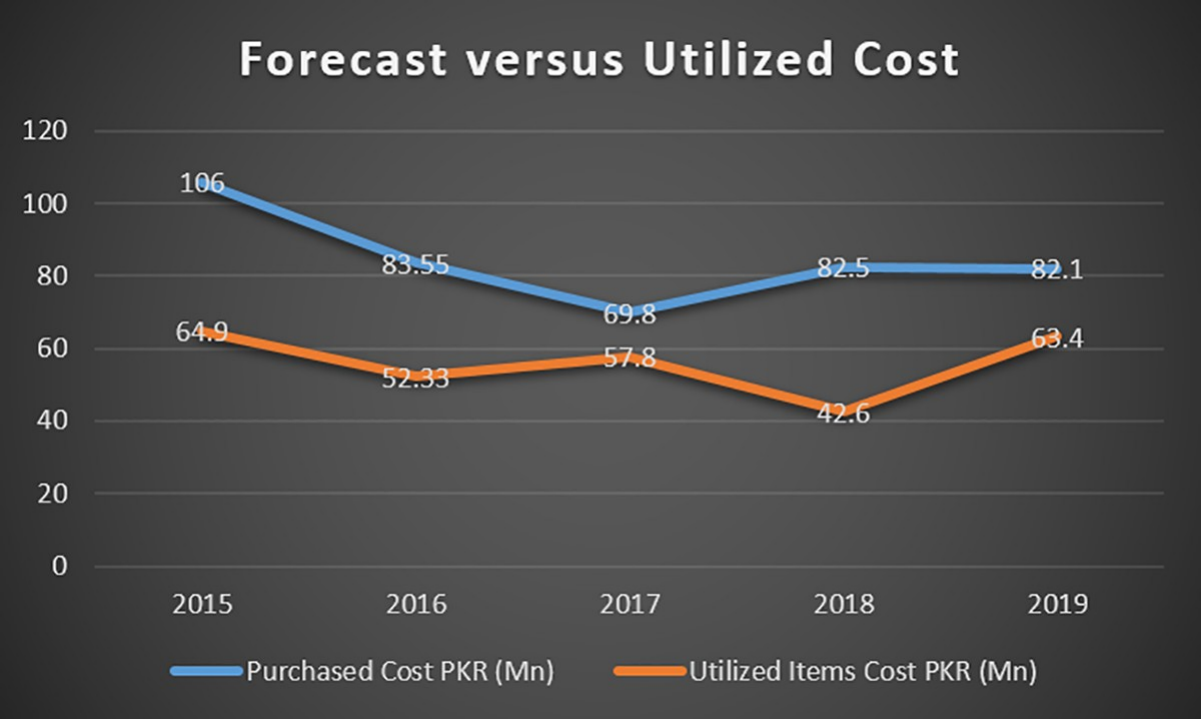
Forecast cost versus utilized cost.

The last five years’ historical data reveals a significant gap between the projected and demanded quantities of parts. Another essential aspect of the data is the erratic behavior and intermittency of the forecasted values for most parts. In 2015 there is only 67% of spares utilization (in terms of quantity), which rises to 152% in 2016 with a gradual drop in 2017 and 2018 and a sudden rise in 2019. A comparison of forecast (purchased cost) and actual demand (utilized cost) for the last five years can be made from the graph in Fig 2 below.

Forecast cost and actual demand cost curves are entirely misaligned, and a considerable difference exists at all instantaneous points. This difference has created a significant value of stuck amount and an upward trend in inactive inventory and dead spares. There is an enormous difference between forecast cost and actually utilized demand cost, which strengthens the fact that the forecast is not based on a systematic procedure or modern techniques. Based on this analysis, the studied organization’s complete retaining stock is segregated into three categories Barrow & Kourentzes [34] shown in Table 3.

**Table 3:** Retaining stock segregation based on analysis of historical demands.

Active Inventory	Items demanded ≥ one time in a year	121
Inactive Inventory	1 ≤ Item demanded ≤ two times in 5 years	1208
Dead Inventory	Items never demanded in the last five years	< 2145

### 4.2. Fault trend analysis (stage-2)

**Fig 3:**
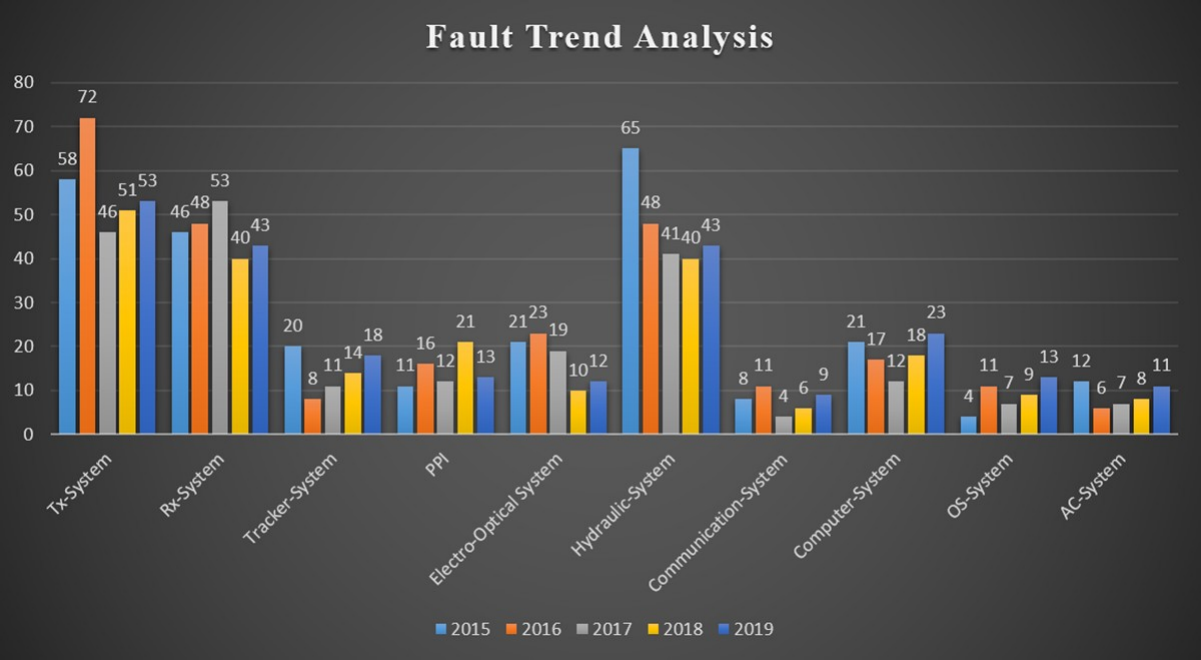
Fault rates in subsystems of surveillance equipment 2011–2015.

In Stage -2, Fault Trend Analysis of different subsystems of Surveillance Equipment being repaired at the studied organization is carried out from 2015 to 2019. Different subsystem varies in their fault rates which are adequately recorded by Planning and Control Department of the studied organization during their repair/maintenance process as shown in Fig 3.

It can be seen from the graph as mentioned above, that there are three subsystems, i.e., Tx System, Rx System, and Hydraulic System, with a maximum number of faults each year. In contrast, Communication System, OS System, and AC System have a minimum number of faults, and the rest of the subsystems lie between these two extremes. Based on the last five years’ fault rates, these subsystems can further be segregated into three categories, as shown in Table 4 below.

**Table 4:** Categorization of subsystems based upon annual fault rates.

Category-1	Fault rate ≥ 40 per year	Tx System, Rx System, Hydraulic System
Category-2	10≤ Fault rate≤ 20 per year	Tracker System, PPI, EO System, Computer System
Category-3	Fault rate ≤10 per year	Communication System, OS system, AC system

A significant chunk of spares parts of active inventory belongs to Category-1 subsystems. About 64% of faults are related to the category-1 subsystems, whereas only 36% belong to the rest of the seven subsystems, which indicates that most of the active inventory items belong to category-1. These "Active non-repairable items" are required to be forecasted in a systematic way to avoid wrong purchasing and compilation of dead inventory spare parts which can be repaired at the organizational level and need not to be replaced with a new one are not considered in this study as these spares are not part of the forecasting process and can easily be repaired through replacement of minor components.

### 4.3. Formulation of comparative analysis matrix (stage-3)

Some spares are cheaper than others, whereas other spares are either expensive or highly expensive. Similarly, some parts are kept in low quantities, and some are kept in larger quantities based upon their usage rate and historical data trend. Costly spares create the most significant and essential impact as they utilized the annual budget’s main chunk. Forecasting of these spares is of extreme importance as it has a direct impact on the overall budget and can have severe repercussions if not purchased judiciously; on the other hand, spare parts which are usually required in large volume are also crucial for forecasting as they also have a significant accumulated impact on the annual budget of the spares. To resolve this issue systematically (3 x 3 Price / Quantity), comparative analysis matrices have been formulated. The primary purpose of these matrices is to isolate *critical items* in terms of price and quantity. All those spare parts segregated as the most critical items in terms of price and quantity represent the significant portion of the annual budget. Two types of categories are formed while formulating the comparative analysis matrix. One is the "Price based category," and the other is the "Quantity based category." This categorization’s basic idea is to select and analyze 121 active inventory parts, which are further distributed in nine boxes of the matrix for further detailed analysis.

#### 4.3.1 Price based analysis

In Price Based Analysis, all active inventory items are divided into three subcategories based on their price, i.e., low (Lo≤ 0.1 Mn), Medium (0.1 Mn ≤ M ≤ 0.5 Mn), and Higher (H ≥ 0.5 Mn). Similarly, the quantity based category is also divided into three subcategories, i.e., small (S≤ 100 units), Medium (100 units≤ M≤150 units), and large (L≥150 units), as shown in Table 5 below.

**Table 5:** Price based analysis.

Category	Subcategory	No. of parts	Total No. of parts	No. of units	Total Nos of units	Price	Total Price	Quantity	Price
						(Mn)		(%)	(%)
**LOW PRICE**	LoS	74	**92**	3526	**6407**	**0.795**	**1.485**	**81%**	**8%**
	LoM	9		1161		**0.41**			
	LoL	9		1720		**0.280**			
**MEDIUM PRICE**	MS	10	**13**	430	**817**	**1.03**	**4.65**	**10%**	**27%**
	MM	1		129		**1.09**			
	ML	2		258		**2.53**			
**HIGH PRICE**	HS	16	**16**	688	**688**	**10.98**	**10.98**	**9%**	**65%**
	HM	0		0		**0**			
	HL	0		0		**0**			

It is quite evident from the above analysis that although low price items comprise a significant portion of active inventory (81% in terms of quantity), yet they have the lowest cumulative effect on the overall inventory annual budget (i.e., 8% of the total expenditure). In contrast, only 16 parts (9% in terms of quantity) fall in the high price parts category, but they have the most significant impact (i.e., 65% of the total expenditure). Therefore, it can be deduced that particular importance should be given to forecasting high price & small quantity (HS category) spares.

#### 4.3.2 Quantity based analysis

In Quantity Based Analysis, the active spares are again distributed based on predetermined criteria amongst nine boxes (subcategories) of 3x3 matrices, as shown in Table 6 below.

**Table 6:** Quantity based analysis.

Category	Subcategory	No. of part	Total No. of parts	No. of units	Total Nos of units	Price	Total Price	Quantity	Price
						(Mn)		(%)	(%)
							(Mn)		
**SMALL**	SLo	74	**100**	3526	**4644**	**0.795**	**12.805**	**60%**	**75%**
	SM	10		430		**1.03**			
**QUANTITY**									
	SH	16		688		**10.98**			
**MEDIUM**	MLo	9	**10**	1161	**1290**	**0.41**	**1.5**	**16%**	**9%**
	MM	1		129		**1.09**			
**QUANTITY**									
	MH	0		0		**0**			
**LARGE**	LLo	9	**11**	1720	**1978**	**0.280**	**2.81**	**24%**	**16%**
	LM	2		258		**2.53**			
**QUANTITY**									
	LH	0		0		**0**			

As per Quantity Based Analysis, small quantity Items are around 60% of the total quantity and consume 75% of the overall inventory budget. Within the small quantities items, high price items carry the significant chunk. Quantity-based analysis re-emphasized the deduction made through price-based analysis to closely track and continuously monitor ***high price and small quantity*** parts **(HS category)** since they constitute the significant portion of the annual spare budget.

### 4.4. Determination of "optimum retention inventory stock" (stage-4)

Active spares have already been identified in the first three stages of DSS. Stage-4 uses three different forecasting methods, i.e., Weighted Moving Average, Exponential Smoothing, and Trend Projection, to predict the required quantity of these spares.

**Fig 4:**
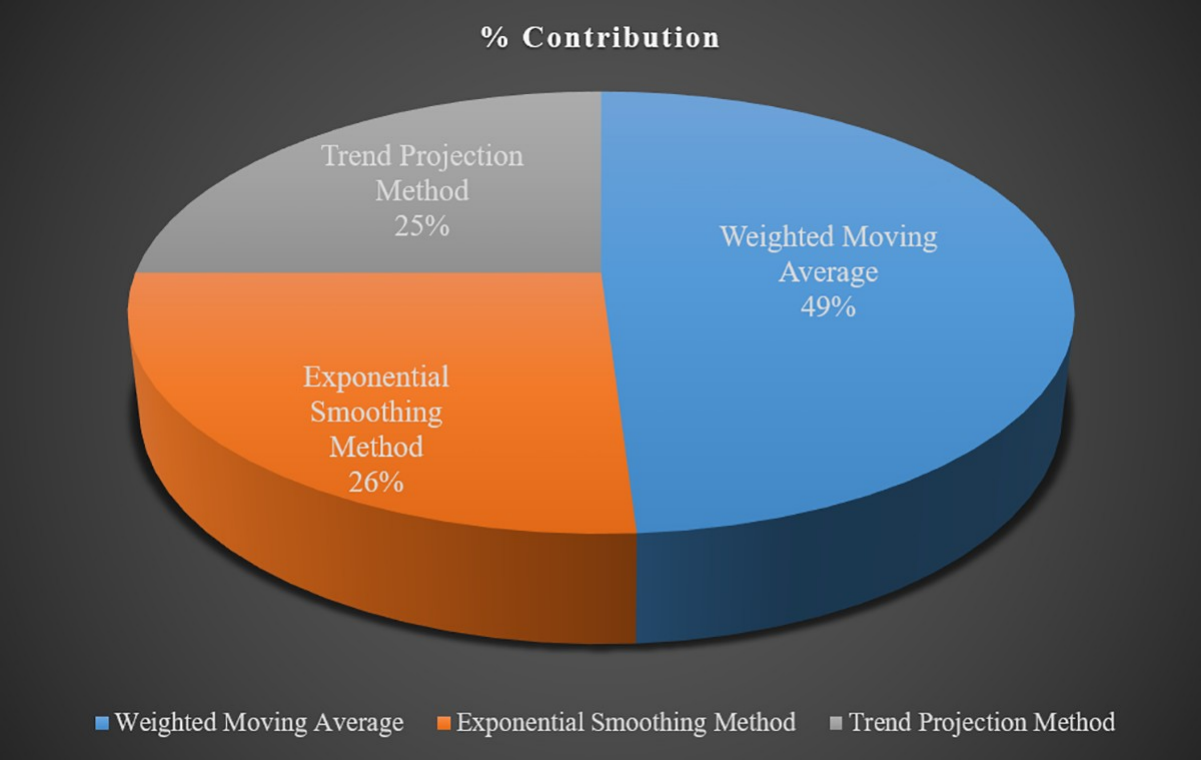
% Contribution of three forecasting methods.

Moreover, some essential forecasting factors are also considered carefully to predict with minimum demand errors. There are definite trends in specific demands, which are denoted by an upward or downward slope. Seasonality is another crucial factor that is considered a data pattern that repeats itself over 1 to 2 years. Due to some extraordinary events, certain irregular fluctuations can also be observed in spares demand patterns. Sometimes these variations are incredibly random and need to be smoothened effectively, and sometimes a smooth trend line makes it easy to forecast demand in the right quantity. On the other side, the forecasting approach’s performance is measured through "Accuracy," i.e., the closeness of forecasted values to the actual values. In this research work, "Forecasting Errors" are calculated using "Mean Absolute Deviation (MAD)" and "Mean Absolute Percentage Error (MAPE)" methods. Both of these methods are globally accepted and reliable. The forecasting method with minimum MAD and MAPE value is selected out of three. It proved to be a straightforward and easy-going approach without compromising on accuracy, as shown in Fig 4.

**Fig 5:**
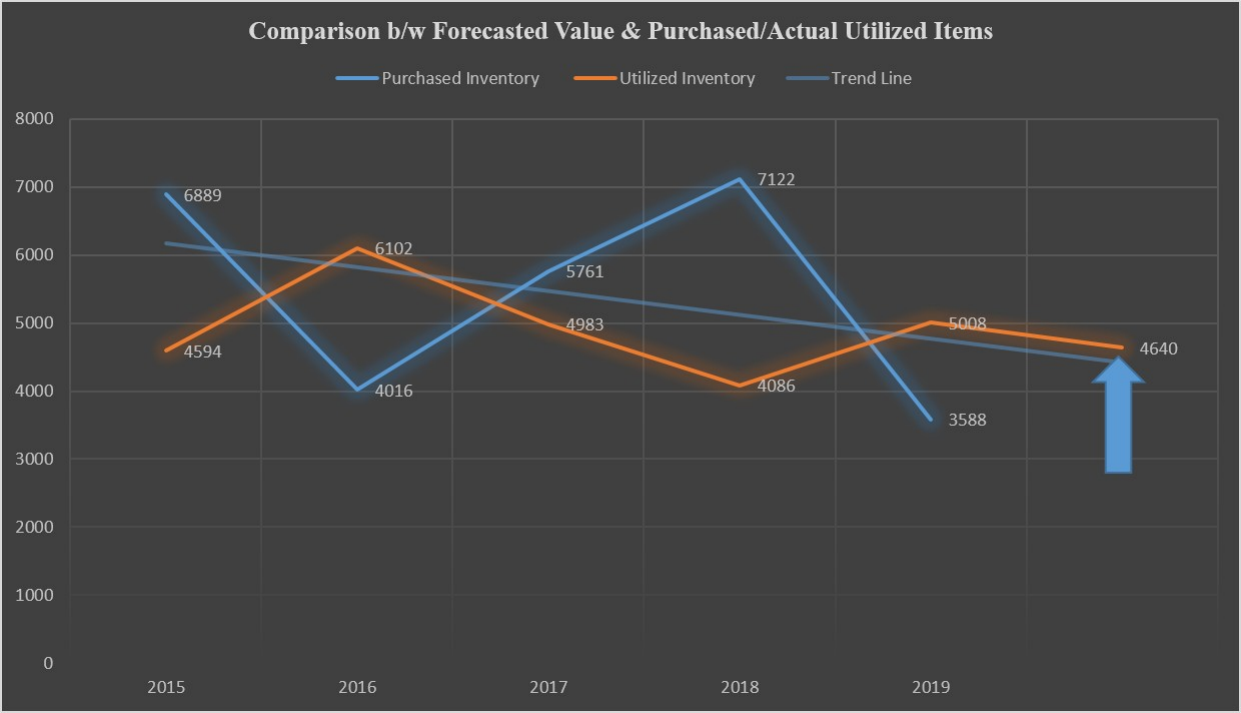
Comparison of forecasted value with purchased/actual utilized items.

Results show that 49% of the contribution is made by the Weighted Moving Averages method; 26% of the contribution is made by the Exponential Smoothing method, whereas 25% of the contribution is made by the Trend Projection method. A total of 4640 units of 121 active spares have been forecasted and plotted in Fig 5 below to compare with historical forecasts.

## 5. Results discussion

The first step of the study was to assess the last five years of forecasted data and utilize spares to have a fair idea of the demand-supply curve and estimate potential forecast errors. As the results (see Table 1) show, there is a significant gap between projected values and actual spares’ consumption each year. The historical data also reveals the erratic behavior and intermittency of the forecasted values for most inventory items. Such kind of forecast anomalies is generally caused by; (1) non-consideration of historical demands record while forecasting the future demands, (2) non-consideration of trend projections, (3) over-reliance on personal experiences instead of statistics and quantitative findings, (4) non-utilization of established forecasting techniques (5) bulk-purchasing, etc. [1, 4]. The next step was to classify the retention stock/holding inventory into the active, inactive, and dead inventory through statistical assessment as per the laid down criteria [34]. This classification (Table 3) helps organizations; (1) to maintain control over the critical items with vast sums of capital invested in them (2) to ensure that the inventory turnover ratio is methodically maintained at a reasonably higher level through systematic control of holding stock (3) to make sure optimum levels of inventory is preserved at all times [23]. At stage-2, the outcomes of fault trend analysis (Fig 3) are used to determine the frequencies of multiple faults to identify the related spares requirement and inventory trend.

Faults are defects in the production process that are generally caused by (1) substandard spares, (2) low skill level of technicians/workforce, (3) inappropriate tools and techniques, and (4) inadequate process management [28, 30, 34]. Bacchetti & Saccani [17] argued that fault trend analysis and their projective manifestations are useful in every production process to avoid fundamental causes and demand uncertainties and future project outcomes. Thus, all manufacturing and engineering organizations (irrespective of their size) must utilize suitable fault/failure rate analysis techniques and ensure better record keeping of fault trend data. At stage-3, the 3x3 (Tables 5 & 6) comparative analysis matrix technique yields the most relevant chunk of inventory that has to be taken care of while forecasting future demands. As stated by the famous Pareto principle, *"80% of consequences come from 20% of the causes*, *asserting an unequal relationship between inputs and outputs"* [35]. It is the case with the outcomes of price-based and quantity-based analysis, which indicate that a small amount of High Price and Small Quantity items (HS items) consumes the main chunk of the inventory procurement budget. The procurement department has to be on a *"high alert"* while forecasting these items. Bulk procurements may be acceptable for a low price and large quantity items, while JUST IN TIME is a plausible solution for HS inventory as it makes an intuitive sense. Likewise, the first three stages’ identified active spares require careful forecasting with minimum forecasting errors and severe consideration of factors like demand patterns, trends, and seasonality [7]. No single forecasting method can predict the entire retention stock precisely. Different methods are useful for different data patterns.

In some cases, due to some extraordinary events, certain irregular fluctuations can be observed in spares demand patterns. Sometimes these variations are incredibly random and need to be smoothened effectively, and sometimes a smooth trend line makes it easy to forecast demand in the right quantity. Lastly, a comparison of historical data between purchased versus actually utilized items is made to identify the forecasting gaps and errors. The convergence of current forecast demand and trend line (Fig 4) shows optimum prediction results. Nevertheless, each stage of the current framework has lessons for organizations to learn; to improve production, achieve cost-effectiveness, avoid demand uncertainty and maintain better inventory control.

## 6. Implications

During the Fourth Industrial Revolution, the notion of inventory leanness has evolved as a critical concern for engineering and manufacturing organizations worldwide [3, 36]. In Pakistan, there are about two hundred Public Sector’s TSOs [37]. These Non-Profit (Government) TSOs consume about the US $2.9 billion of the annual budget [38] to provide technical expertise like manufacturing, repairs, maintenance, and modifications to all electrical fields mechanical, optronics, surveillance, and IT equipment of defense forces. The findings of the current study provide useful insights and guidelines to all such organizations. It explains how the inventory leanness can be achieved by applying this hybrid framework to avoid heavy consumptions on dead inventories, eliminating waste, and achieving cost-effectiveness. The practitioners and managers of other TSOs can replicate this novel approach to save a significant amount of procurement, further enhancing their product quality, on-time delivery, customer satisfaction, preventive maintenance, etc.

The DSS with minor modifications can also be utilized by those manufacturing organizations involved in annual/bulk procurements (due to lead-time/policy issues) to handle regular and erratic demands. Replacing fault trend with failure rate/cause analysis in the current framework can help manufacturing organizations to improve product design, the mean time before failure (MTBF), and maximizing Machinery Uptime.

Likewise, one of the most critical problems the manufacturing SMEs face in Pakistan is poor inventory management and control due to ineffective inventory forecasting, inadequate record-keeping, and misalignment of sales and demand curves [39, 40]. Abbas [41] also indicated that lack of inventory management expertise had hindered small and medium-sized manufacturing companies from becoming powerful rivals in the manufacturing sectors. The Proposed DSS can help SMEs maintain an adequate inventory record using this study’s inventory classification criteria. They also have useful lessons to learn from price and quantity based comparative analysis matrix to align their sales and inventory forecasts.

Lastly, irrespective of the regional boundaries and industries, the current framework can be a useful tool for better inventory management and control, especially when dealing with erratic inventory demands.

## 7. Conclusion

The proposed DSS sets a solid foundation for manufacturing organizations in general and TSOs to handle critical issues like overbuying, under productions, stock out situations, unexpected delays in raw material deliveries, and retention stock discrepancies. It offers a plausible solution to find the answer to two critical questions (1) *what is to be procured* (2) *how much to be procured*? to maintain the "Optimal Retention Inventory Stock." The DSS provides a robust and systematic solution for identifying the active inventory stock and determining the "*most critical items*" in terms of price and quantity. It offers a sequential decision-making process to single out the "*high price and a small quantity* (HS)" items to handle significant contributors to the procurement budget safely. The proposed DSS further estimates the suitable procurement volume of inventory to avoid excess purchasing and compilation of dead inventory items through minimum error calculations with multiple techniques. Although the DSS is applied explicitly to a Public Sector’s Technical Services Organization as a case study, it has the flexibility to be applied to manage service parts in a wide variety of environments.

## 8. Limitations and future research

Despite its effectiveness in various facets of inventory replenishment and control, there are various limitations associated with the current study. Regardless of its robustness, the current DSS is lengthy, and time/efforts are consuming. The development of DSS software while incorporating all the sequential stages of the current DSS can significantly reduce the time/effort aspects. An effective forecast of intermittent and lumpy demand is a challenging aspect. Demand occurs only sporadically and, when it does, it can vary considerably. Forecast errors are costly, resulting in obsolescent stock or unmet demand. Methods from statistics, machine learning, and deep learning need to be incorporated into the current DSS to predict such demand patterns more efficiently.
